# Functional Regulation of Macrophage Phenotypes by MicroRNAs in Inflammatory Arthritis

**DOI:** 10.3389/fimmu.2019.02217

**Published:** 2019-09-13

**Authors:** Xiaoling Niu, Grant S. Schulert

**Affiliations:** ^1^Department of Nephrology and Rheumatology, Shanghai Children's Hospital, The Children's Hospital of Shanghai Jiaotong University, Pudong, China; ^2^Division of Rheumatology, Cincinnati Children's Hospital Medical Center and Department of Pediatrics, University of Cincinnati College of Medicine, Cincinnati, OH, United States

**Keywords:** macrophage polarization, rheumatic arthritis, juvenile idiopathic arthritis, systemic juvenile idiopathic arthritis, microRNA mimics, microRNA inhibitors

## Abstract

Inflammatory arthritis including rheumatoid arthritis (RA) and juvenile idiopathic arthritis (JIA) exhibit the shared feature of changes in activation and polarization of circulating monocytes and tissue macrophages. Numerous microRNAs (miRs) have been found to have key functions in regulating inflammation and macrophage polarization. Although there is increasing interest in the roles of miRs in both RA and JIA, less is known regarding how miRs relate to functional properties of immune cells, including monocytes and macrophages. Interestingly, miRs can function both to promote inflammatory phenotypes and pro-inflammatory polarization, as well as through negative-feedback loops to limit inflammation. Here, we review the functional roles of several miRs in macrophages in inflammatory arthritis, with a particular focus on vivo effects of miR alteration in experimental arthritis. We also consider how current efforts to target miRs clinically could modify functional monocyte and macrophage polarization *in vivo*, and serve as novel therapies for diseases such as RA and JIA.

## Introduction

Rheumatoid arthritis (RA) is a systemic autoimmune disease with risk of functional disability due to articular damage as a consequence of ongoing inflammation. Patients with RA exhibit an inflammatory environment that favors the activation of neutrophils, T cells, B cells, macrophages, osteoclasts, and synovial fibroblasts (1). Juvenile idiopathic arthritis (JIA) is the most common rheumatologic condition in children, and encompasses a group of disorders with the shared feature of chronic arthritis with or without other symptoms. According to the current classification by the International League of Associations for Rheumatology (ILAR), JIA consists of 7 categories (2), several subtypes of which have strong phenotypic and pathologic similarity to forms of arthritis including RA, while others represent distinct entities (3). Systemic JIA (sJIA) is a distinct auto-inflammatory disease, characterized by systemic inflammation, with risk for severe complications including macrophage activation syndrome (MAS) (4). MAS results from over-activation of T lymphocytes and macrophages leading to a life-threatening “cytokine storm” (5). While these various forms of inflammatory arthritis have key genetic and pathologic differences, they exhibit the shared feature of changes in activation and polarization of monocytes and macrophages.

Macrophages display distinct functional differences dependent on their micro-environments through a process referred to as polarization. Historically, activated macrophages have been considered to have two broad phenotypes: M1 (classically activated) macrophages and M2 (alternatively activated) macrophages. M1 macrophages are induced by bacterial products such as lipopolysaccharide (LPS), tumor necrosis factor (TNF), and interferon (IFN)-γ secreted by Th1 cells, and can phagocytose directly and kill pathogenic microorganism and tumor cells. M2 macrophages are induced by steroids, IL-4, IL-13, IL-10 and transforming growth factor (TGF)-β secreted by Th2 cells. These generally limit immune response by secreting suppressive cytokines, reducing pro-inflammatory factors, and increasing expression of scavenger receptors which participates in fibrosis (6, 7). M2 macrophages also mediate humoral immunity, tissue repair, vascularization, and tumor promotion/invasion (6, 8).

Macrophages are famous for their phenotypic heterogeneity and the diverse activities. Indeed, recent work has shown that M1 vs. M2 model of macrophage polarization is a marked oversimplification, and these cells rather have a plastic gene expression signature that is influenced by the type, concentration, and duration of exposure to different activating stimuli (9). That diversity allows monocytes and macrophages to serve important roles in the development of several autoimmune and auto-inflammatory diseases including inflammatory arthritis. Transcription factors are key molecules for determining the functional polarization in macrophages (10–12). These transcription factors are regulated themselves transcriptionally, post-transcriptionally, and at the protein level, and increasingly microRNAs (miRs) are included in that which can regulate transcription factors.

MiRs are endogenous small non-coding RNAs that induce inhibition of target gene expression by binding to direct complementary sequences in the 3′ untranslated region of mRNAs (13). MiRs are regulatory molecules involved in many physiological processes, including cellular differentiation and immune cell activation. MiRs regulation is characterized by its active participation in and strict control of the negative feedback loop to “fine tune” gene expression programs (14).

Over the past decade, there are many studies of miRs in patients with RA and JIA. This work has identified altered miR expression levels, particularly in peripheral blood. But less is known regarding how miR relate to functional properties of immune cells in arthritis, such as monocytes and macrophages. Here, we will review the functional roles of miRs in macrophages in RA and JIA, with a particular focus on *in vivo* effect of miR alternation in experimental arthritis.

## MicroRNA-155

MicroRNA-155 is a multifunctional miR enriched in cells of the immune lineage. MiR-155 is encoded within a B cell integration cluster gene, and can be induced by pro-inflammatory ligands such as LPS and TNF (15–17). It plays important roles in arthritis by regulating the polarization of macrophages, cytokine and chemokine production, and resistance to apoptosis. MiR-155 was significantly higher in blood monocytes from RA patients, and levels correlated with disease activity measures including the disease activity score (DAS)-28 and erythrocyte sedimentation rate (ESR) (18, 19). It was also increased in fibroblast-like synoviocytes in RA patients compared to healthy controls and patients with osteoarthritis (OA) (20). MiR-155 was also increased in plasma of JIA patients, but cellular levels were not determined (21). Recent work has found that miR-155 is increased in monocytes from children with active sJIA compared to controls or clinically inactive sJIA (22).

**Figure 1:**
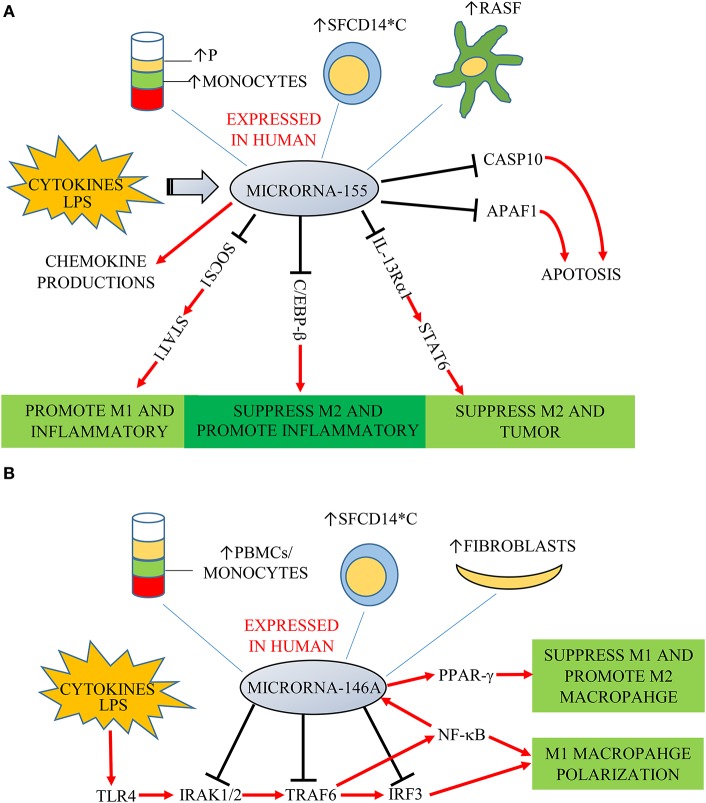
**(A)** MiR-155 is expressed in plasma (P), monocytes, fibroblast-like synoviocytes (RASF) and synovial fluid monocytes (SFCD14*C) of patients with RA/JIA. It is induced by cytokines and LPS, and overexpression increases chemokine production. SOCS1, IL-13Rα1, and C/EBP-β are key target genes of miR-155. SOCS1 is a negative regulator of STAT1. MiR-155 decreased SOCS1 expression, increasing signaling through STAT1 to promote M1 macrophages and suppress M2 macrophages to promote inflammatory responses. MiR-155 could also directly target C/EBP-β to suppress M2 macrophages. MiR-155 directly targets IL-13Rα1 and decreases the levels of IL-13Ra protein, resulting in decreased activation of the M2-promiting STAT6. MiR-155 is also associated with decreased expression of two predicated miR targets that mediate apoptosis: CASP10 and APAF1. **(B)** MiR-146a was expressed in PMBCs, monocytes, synovial fibroblasts, and synovial fluid monocytes (SFCD14*C) of patients in RA/JIA. It is induced by cytokines and LPS through the NF-κB pathway. It controls TLR4 signaling through a regulatory loop: the upregulation of miR-146a by caused by activated NF-κB; miR-146a reduces the expression of its targets including TRAF6, IRAK1, IRAK2, and IRF3; which limits activity of both NF-κB and IRF3 pathways.

Functionally, key target genes of miR-155 include suppressor of cytokine signaling 1 (SOCS1), interleukin 13 receptor α1 (IL-13Rα1) and CCAAT-enhancer-binding proteins (C/EBP)-β (Figure 1A) (23). MiR-155-deficient murine RAW264.7 macrophages and human macrophages gene-silenced for miR-155 express decreased levels of pro-inflammatory cytokines (24, 25). Elmesmari et al. found that miR-155 also regulated chemokine production and pro-inflammatory chemokine receptor expression (18). MiR-155 can broadly promote macrophage M1 polarization and suppress M2 features. SOCS1 is a negative regulator of signal transducers and activators of transcription-1 (STAT1), which mediates signaling from pro-inflammatory cytokines including type I and II IFN (26). MiR-155 decreased SOCS1 transcription by directly targeting its 3′UTR region, thereby increasing pro-inflammatory cytokine and surface molecule expression (27). Besides miR-155 promoting M1 macrophages by targeting SOCS1, it can also suppress M2 macrophages to promote inflammatory responses. Classically, M2 macrophages can be induced by IL-4 and IL-13. Martinez-Nunez et al. demonstrated that miR-155 directly targets IL-13Rα1 and decreases the levels of IL-13R a protein, resulting in decreased activation of the M2-inducing STAT6 in human macrophages from healthy donors (28). Through these mechanisms, miR-155 was also found as a pivotal regulator of M1 inflammatory macrophage signature (29).

Monocytes from peripheral blood of RA and JIA patients are resistant to spontaneous apoptosis, which may lead to persistence of inflammatory monocytes and/or macrophages thereby perpetuating joint inflammation (30). Rajasekhar et al. found that increased mature miR-155 in CD14+ monocytes was associated with decreased expression of two predicted miRs targets that mediate apoptosis: caspase 10 (CASP10) and apoptotic protease activating factor-1 (APAF1). Similarity, overexpression of miR-155 in monocytes from RA patients conferred enhanced resistance to spontaneous apoptosis (30).

Several studies of experimental arthritis in mice have examined the *in vivo* function of monocyte and macrophage miR-155 expression. MiR-155 deficient mice have significantly reduced signs of arthritis in the collagen-induced arthritis (CIA) model (24). In support of this, miR-155 deficient mice are also protected from experimental colitis. In this system, miR-155 knock-out macrophages exhibit an M2 phenotype, and depletion of these macrophages reconstitutes colitis (31). On the other hand, miR-155 was recently found to be dispensable for urate-induced arthritis, suggesting its effect may be context-specific (32).

## MicroRNA-146a

MicroRNA-146a plays a critical role as a regulator of innate immune responses. It is located in the second exon of the LOC285628 gene on chromosome 5 and is generated in response to inflammatory stimuli such as LPS, TNF, IL-1β, or toll-like receptor (TLR) ligands in various cells, particularly in monocytes/macrophages (33). MiR-146a was increased in the peripheral blood mononuclear cell (PBMC), synovial fibroblasts and synovial fluid in RA patients as well as monocytes from children with sJIA (19, 22). Recently, miR-146a-5p was found to be expressed in peripheral monocytes from patients with psoriatic arthritis (PsA) (34).

It has been found that miR-146a expression was an important player in “fine-tuning” the NF-κB signaling pathway in monocytes, as it downregulates key pathway components inducing IL-1 receptor-kinase 1 (IRAK1), IRAK2 and tumor-necrosis-factor receptor associated factor (TRAF)-6 (Figure 1B) (35). MiR-146a was found also to target interferon regulatory factor (IRF)-3, which is another component of the TLR4 pathway. MiR-146a thus controls TLR4 signaling through a regulatory loop: upregulation of miR-146a by activated NF-κB; miR-146a reducing expression of TRAF6, IRAK1, IRAK2, and IRF3 and a resulting reduced activity of both NF-κB and IRF3. However, there is conflicting information regarding the role of miR-146a in monocyte and macrophage polarization. Increased expression of miR-146a was reported in M1 polarized macrophages stimulated by IFN-γ and TNF (36). On the other hand, other studies found miR-146a being higher in M2 polarized human and mouse macrophages, and promoting M2 polarization by targeting inhibin beta A (INHBA) (37, 38). Similarly, Huang et al. found miR-146a could promote M2 macrophage polarization by enhancing the activation of PPAR-γ in RAW264.7 cells (39).

Several models of experimental arthritis has shown that miR-146a expression has key roles in modulating disease *in vivo*. Expression of miR-146a inhibits osteoclastogenesis and administration of double-stranded miR-146a prevents joint destruction in CIA mice (40). Similarly, mice with miR-146a deficiency develop more severe gouty arthritis (41) and show increased articular inflammation in lyme arthritis (42). Some recent work suggests that these effect of miR-146a may be monocyte-mediated. Specific miR-146a overexpression in Ly6C^hi^ monocytes decreased signs of bone damage in CIA mice (43). Together, this suggests miR-146a as a novel therapeutic target for bone destruction in inflammatory arthritis.

## MicroRNA-let7a

MicroRNA-let7a belongs to let-7 family and known to be involved in inflammation and cellular apoptosis. MiR-let7a is highly expressed in human macrophages and it can directly target Ras expression (44). Several studies have examined the role of miR-let7a in RA, demonstrating that miR-let7a was decreased in monocytes and synovial fluid macrophages from anti-citrullinated protein antibody (ACPA)-positive RA patients (45, 46). Indeed, Lai et al. found ACPAs suppressed miR-let7a expression levels in monocytes from ACPA-positive RA patients, and contributed to the pathogenesis of RA. ACPAs could induce phosphorylation of extracellular signal-regulated kinase1/2 (ERK1/2) and c-Jun N-terminal kinase (JNK), which were downstream mediators of the Ras signaling pathway. However, after transfection with miR-let7a, ACPAs failed to do that. They also found that transfection of miR-let7a could suppress the mRNA and protein expression of IL-1β (45). There are no reports about the miR-let7a in macrophage function and polarization in JIA. In this regard it is notable that children with JIA rarely have ACPA present.

Despite these findings, there is less evidence that macrophage miR-let7a expression alone is sufficient to drive experimental arthritis Although Zhang et al. found that miR-let7a overexpression decreased macrophage infiltration into tumors (47), Zhu et al. demonstrated that miR-let7a agomir could not mitigate the development of CIA in mice (46). More research in experimental arthritis models are needed to clarify whether miR-let7a represents an attractive therapeutic target.

## MicroRNA-33

MicroRNA-33 is located in introns of the sterol regulatory element-binding protein (SREBP)-encoding genes and controls cholesterol/lipid homeostasis in concert with their host gene products. It plays a key role in atherosclerosis and mediates regulation in the metabolic pathways, inflammatory response, insulin signaling and glucose homeosstasis (48). Recent work has found that both miR-33 and nucleotide binding domain and leucine-rich repeat pyrin 3 domain (NLRP3) inflammasome activity were increased in monocytes from RA patients (49). This miR was reported to regulate macrophage cholesterol by targeting the lipid efflux transporters ATP binding cassette (ABC) A1 and ABCG1 (50–52). MiR-33 also impaired mitochondrial oxygen consumption rates, resulting in the accumulation of cellular ROS, which stimulated NLRP3 expression, CASP1 activity and IL-1β secretion (49). It was also found that miR-33 regulated macrophage inflammation and reduced plaque inflammation by promoting M2 macrophage polarization and Treg induction (53).

As such, the role of miR-33 *in vivo* requires further exploration but many represent an intriguing target. MiR-33 was found to suppress CCL2 levels in the supernatant of cultured primary mouse chondrocytes and miR-33 deficient chondrocytes potentiated monocyte chemotaxis in a CCL2 dependent manner, providing a potential mechanism of macrophage infiltration in OA (54). Macrophages treated with anti-miR-33 also showed increased efferocytosis, lysosomal biogenesis, and degradation of apoptotic material; and treating atherosclerotic Ldlr-/- mice with anti-miR-33 restores defective autophagy in foam cells and plaques (55).

## MicroRNA-125a

MicroRNA-125a is a homolog of *C. elegans* lin-4, is encoded as part of the miR-99b/let-7e/125a miRs cluster and shares high homolog to miR-125b. It contributes to the control of phase transitions in development and/or cell differentiation, regulates the expression of several target proteins that involved in cell proliferation, apoptosis and migration, counteracting viral replication (56) and has key roles in macrophage polarization. This miR has also been examined in both RA and sJIA. The combination of three specific miRs, including miR-125a-5p, was proposed to be used as potential biomarkers for the identification of RA patients (52). In sJIA, miR-125a-5p was highly upregulated in monocytes from children with active disease, as compared to those with inactive disease, and correlated with systemic features of the disease (22).

Expression of miR-125a is induced by TLR signaling through direct NF-κB activation, and is also increased in several alternatively activated macrophage subsets (57, 58). These contradictory finding likely reflect different in species and macrophage type. In mice, miR-125a targets the transcription factor Kruppel-like factor (KLF)-13 to suppress classical macrophage activation and promote an M2 phenotype. But other studies reported miR-125a targeted IRF4 to promote M1 and inhibit M2 polarization in tumor associated macrophages (59). MiR-125a was also upregulated and essential for monocyte to osteoclast differentiation *in vitro* (58). *In vitro*, overexpression of miR-125a-5p in macrophages altered polarization phenotypes toward M (LPS+IC) or “M2b,” which closely resembles that seen in monocytes from sJIA. Further work found that miR-125a expression restricts other polarization phenotypes, including limiting expression of the anti-inflammatory scavenger receptor CD163 (60). However, to date there is little data regarding.

Whether miR-125a-mediated maintenance of immuno-regulatory monocytes and macrophages can be an effective strategy for altering inflammation *in vivo*.

## MicroRNA-223

MicroRNA-223 is located on the X chromosome (61) and is highly expressed in the myeloid compartment and has effects on cell differentiation, inflammation, and oncogenesis (62). There are conflicting reports regarding serum levels of miR-223 in arthritis, with one study showing reduced levels in patients with early RA compared to those with established RA (63). On the other hand, in JIA patients, plasma levels of miR-223 were significantly higher than controls (64). Substantial work has also examined functions of miR-223 in the inflammatory synovium. One report found miR-223 was more highly expressed in RA synovium than in OA patients due to the increased number of miR-223-positive cells (65). This may reflect different underlying causes of arthritis in patients.

Broadly, miR-223 is involved in the polarization and activation of macrophages. MiR-223 promotes the polarization of macrophages toward M2 macrophage phenotype by direct targeting of PBX/Knotted 1 Homeobox 1 (Pknox1) (66). The expression of miR-223 is reduced in macrophages during inflammation due to TLR ligand stimulation (66, 67). MiR-223 can also control macrophage inflammatory responses by inhibition of NLPR3 inflammasome activity (68).

In support of a key role for miR-223 in arthritis pathogenesis, prior work has demonstrated miR-223 overexpression in both RA patients and CIA mice synovium, and that down-regulation of miR-223 in mice reduced multiple markers of disease activity (69). Moriya et al. also demonstrated that miR-223 was increased in SKG mouse plasma (70). Finally, recent work found that microovesicles containing miR-223 (and miR-142) selectively targeted lung macrophage and reduced tissue responses (71). Taken together, miR-223 has multiple functions on the polarization and activation of macrophages with relevance *in vivo* or in vitro to RA and JIA.

## Other microRNAs in Inflammatory Arthritis

There are several reports of additional functional miRs in macrophages in RA and JIA. MicroRNA-124 is a critical modulator of immunity and inflammation (72). MiR-124 was reported to promote M2 polarization and attenuated inflammatory response by targeting C/EBP-α (73). It also ameliorated adjuvant-induced arthritis (AIA) in mice by targeting critical mediators of arthritis development, such as receptor activator of nuclear factor-kappa B ligand (RANKL) and nuclear factor of activated T-cells, cytoplasmic 1 (NFATc1) (74). MicroRNA-26a has important roles in the progression of bone damage and repair, osteoblast differentiation, lipid metabolism, and tumor biology (75). It can promote M1 macrophage polarization and suppress M2 by targeting KLF4 and MYC binding protein (MYCBP) (76, 77). MiR-26a is overexpressed in plasma and PBMCs of RA patients and upregulated in the differentiation of IL-17 producing CD4^+^ cells, which are important for RA pathogenesis (78). It also negatively regulates TLR3 signaling via targeting of TLR3 itself in rat macrophages, and reduced disease severity in pristine-induced arthritis (79). MicroRNA-19a and microRNA-20a are members of microRNA-17-92 cluster and involved in carcinoma, hypoxia, stem cell regulation, and monocyte-to-macrophage differentiation (80). MiR-20a could regulate ASK1 protein expression and TLR4-dependent cytokine release in rheumatoid fibroblast-like synoviocytes (81). MiR-19a and miR-21 showed reduced expression in PBMCs in sJIA, while mRNA of predicted targets including STAT3, SOCS1, TNF were elevated suggesting a role in JAK/STAT signaling pathways (82). However, in purified monocytes several members of the miR-17-92 cluster were found to be elevated during active sJIA (22), and as such the role of this cluster remains unclear. *In vivo* however, inhibitors of miR-17-92 cluster members reduced thioglycollate-elicted peritoneal macrophage infiltration and phagocytosis in mice (83).

## Conclusion—MIRS as Central Regulators of Macrophage Phenotypes

MiRs are increasingly recognized for their involvement in autoimmune diseases including RA and JIA. Macrophages display diverse functional properties in response to different inflammatory microenvironments. Although this phenotypic complexity is much more extensive than prior models of classical M1 vs. alternative M2 polarization (9), the imbalance of macrophage polarization is a key aspect of RA and JIA pathophysiology (84). MiRs have key roles in regulating macrophage polarization, particularly through targeting multiple transcription factors to both actively suppress certain phenotypes and function as negative feedback loops. For example, miR-155 utilizes multiple mechanisms to both promote M1 phenotypes and suppress M2 features (Figure 1A). On the other hand, miR-146a is induced by pro-inflammatory signals and functions to limit or “fine-tune” TLR4 signaling, thereby dampining classical macrophage polarization (Figure 1B). We have examined miRs associated with inflammatory arthritis and data related to largely *in vitro* effects on macrophage biology (Table 1). Despite sometimes extensive functional data, in general less is known regarding whether these roles translate *in vivo*. In particular, further research regarding cell-type specific effects of miRs *in vivo* are needed to validate the functional roles of these regulators.

**Table 1:** MicroRNAs in macrophages and in RA/JIA.

**microRNA**	**Site of expression**	**Targets**	**Functions**	**Arthritis-specific findings *in vivo***
miR-155	P, PBMCs, SFCD14*c, RASF	SOCS1 IL-13Rα1 C/EBP-β	Promote M1 (26, 27) Suppress M2 (28) Suppress M2 (23)	In CIA (24) In urate-induced arthritis (32)
miR-146a	PBMCs, SFTc, SFCD14*c	IRF3, TRAF6, IRAK1/2INHBA	Regulate polariztion of macrophages (35) Promote M2 (37, 38)	In CIA (40, 43) In gouty arthritis (41) In lyme arthritis (42)
miR-let7a	PBMCs, SFCD14*c, RASF	Ras HMGA2	Suppress inflammation (45) Downregulate macrophage activation (46)	In CIA (46)
miR-33	RACD14*c	ABCA1, ABCG1	Increase inflammation (50–52)	Unknown
miR-125a	P, PB	KLF13 IRF4	Promote M2 (58) Promote M1 (59)	Unknown
miR-223	PB, PBMCs, PTc, S, O/O	Pknox1	Promote M2 (66)	In CIA, SKG (69, 70)
miR-124	RASF	C/EBP-α NFATc1	Promote M2 (73) Suppress osteoclasts (74)	In AIA (74)
miR-26a	PBMCs	KLF4MYCBP	Promote M1 (75) Suppress M2 (76)	In pristine-induced arthritis (79)
miR-20a	RAFLS	Ask1	Suppress inflammation (81)	Unknown
miR-19a miR-21	RASF	STAT3 SOCS1	Increase inflammation (82)	Unknown

*P, plasma; S, serum; PB, peripheral blood; PBMCs, peripheral blood mononuclear cells; PRTc, peripheral regulatory T cells; SFCD14*C, synovial fluid CD14^+^ T cells; RASF, rheumatoid arthritis synovial fibroblast; SFTc, synovial tissue; RACD14*c, rheumatoid arthritis CD14^+^ T cells; PTc, peripheral T cells; O/O, osteoblasts and osteoclasts.

Understanding the *in vivo* functions of miRs including mechanisms of miR-transcription factors networks in macrophage polarization helps provide a basis for macrophage-centered therapeutic strategies. Both miR mimics and molecules targeted at miRs (antimiRs) have shown promise in preclinical development in many diseases, particularly in cancers. Several miR-based therapeutics have gone into the clinical trials (85). A miR-29 mimic may be a therapeutic to prevent formation of a fibrotic scar or to prevent cutaneous fibrosis (86). Two clinical trials of a miR-155 inhibitor in patients with lymphomas and leukemia are undergoing (NCT02580552, NCT03713320). Given the central roles of miR-155 in generating pro-inflammatory macrophage responses, such an inhibitor could have promise in rheumatic diseases. One clinical trial of a miR-92a inhibitor is also ongoing in healthy volunteers (NCT03603431). Given the emerging understanding of miRs in macrophage phenotypes and inflammation in RA and JIA, further pre-clinical and animal model work is needed to define how altering monocyte and macrophage phenotypes through miRs can ameliorate inflammatory arthritis.

## Author Contributions

XN wrote the first draft. XN and GS conceived of the review, revised and edited, and approved the final draft.

### Conflict of Interest Statement

GS has received consulting fees from Novartis. The remaining author declares that the research was conducted in the absence of any commercial or financial relationships that could be construed as a potential conflict of interest
